# 17-oxo-DHA displays additive anti-inflammatory effects with fluticasone propionate and inhibits the NLRP3 inflammasome

**DOI:** 10.1038/srep37625

**Published:** 2016-11-24

**Authors:** Chiara Cipollina, Serena Di Vincenzo, Liboria Siena, Caterina Di Sano, Mark Gjomarkaj, Elisabetta Pace

**Affiliations:** 1 Fondazione Ri.MED, via Bandiera 11, 90133 Palermo, Italy; 2 Istituto di Biomedicina e Immunologia Molecolare, Consiglio Nazionale delle Ricerche, Via Ugo La Malfa 153, 90146 Palermo, Italy

## Abstract

Chronic obstructive pulmonary disease (COPD) is characterized by reduced lung function associated with increased local and systemic inflammatory markers, such as TNFα and IL-1β. Glucocorticoids are used to treat this chronic disease, however their efficacy is low and new drugs are very much required. 17-oxo-DHA is a cyclooxygenase-2-dependent, electrophilic, α,β-unsaturated keto-derivative of docosahexaenoic acid with anti-inflammatory properties. We evaluated the action of 17-oxo-DHA alone or in combination with the steroid fluticasone propionate (FP) in peripheral blood mononuclear cells (PBMCs) from COPD patients and healthy individuals exposed to lipopolysaccharide. We show that PBMCs from COPD patients released higher levels of TNFα and IL-1β compared to controls. 17-oxo-DHA displayed strong anti-inflammatory effects. The addition of 17-oxo-DHA in combination with FP showed enhanced anti-inflammatory effects through the modulation of transcriptional and post-transcriptional mechanisms. 17-oxo-DHA, but not FP, was able to suppress the release of mature IL-1β through inhibition of the NLRP3 inflammasome. Furthermore, 17-oxo-DHA inhibited inflammasome-dependent degradation of the glucocorticoid receptor (GR). Our findings suggest that 17-oxo-DHA in combination with FP or other steroids might achieve higher therapeutic efficacy than steroids alone. Combined treatment might be particularly relevant in those conditions where increased inflammasome activation may lead to GR degradation and steroid-unresponsive inflammation.

17-oxo-DHA is a bioactive electrophilic α,β-unsaturated keto-derivative of the omega-3 fatty acid docosahexaenoic acid (DHA) that is endogenously generated by cyclooxygenase-2 (Cox-2) in activated macrophages^1^. When externally administered, 17-oxo-DHA displays anti-inflammatory and cytoprotective actions, by inducing the Nrf2-dependent anti-oxidant response and suppressing NF-κB-dependent inflammatory reactions^1^,^2^. In experimental models of cigarette smoke-induced inflammation, 17-oxo-DHA protects cells from oxidative stress and inflammation, which affect the lung of patients with chronic obstructive pulmonary disease (COPD)^2^,^3^. Biological actions, chemical properties and endogenous origin make 17-oxo-DHA an interesting lead compound for developing new drugs for the treatment of steroid-resistant COPD.

In COPD patients, reduced lung function is associated with increased levels of local and systemic inflammatory markers. Interleukin 1β (IL-1β) and tumor necrosis factor α (TNFα) are primarily involved in the initiation and persistence of inflammation^4^,^5^. These cytokines respond differently to steroid treatment. TNFα release is strongly suppressed by steroids, while IL-1β poorly responds to this class of anti-inflammatory drugs and its high levels are associated with steroid-resistant neutrophilic inflammation typical of COPD patients. Moreover, the interaction of IL-1β and TNFα with cigarette smoke has been correlated with an overall reduced response to steroids^6^,^7^,^8^. Recently it has been reported that elevated airway IL-1β and increased systemic inflammation are predictors of future exacerbation risk in COPD^5^. The release of mature IL-1β is under tight transcriptional and post-transcriptional control. IL-1β is expressed as a 31 kDa inactive precursor, mostly in response to proinflammatory stimuli^9^. Processing of pro-IL-1β into the mature 17 kDa form occurs through proteolytic cleavage. The cysteine protease caspase-1 is a key enzyme in IL-1β processing. Other proteases, including neutrophil elastase, cathepsin G and caspase-8, are able to convert the IL-1β precursor into the mature form^10^. Caspase-1 is expressed as inactive precursor that is activated via auto-proteolitic cleavage^11^. The activation of caspase-1 involves the formation of the inflammasome, a multi-protein complex that contains procaspase-1, the nucleotide-binding domain leucine-rich repeat-containing receptor (NLR) and the apoptosis-associated speck-like protein containing the caspase-recruitment domain (ASC)^12^. Different inflammasomes contain specific NLR proteins, which respond to different stimuli. The NAIP–NLRC4 inflammasome recognizes bacterial flagellin and T3SS rod/needle proteins^13^ while the AIM2 inflammasome senses cytosolic dsDNA^14^. The NLRP3 inflammasome is activated by exogenous and endogenous compounds including crystals (alum, silica, monosodium urate and others), extracellular adenosine triphosphate (ATP), pore-forming toxins such as nigericin^15^ as well as glycolytic inhibitors and metabolic conditions affecting hexokinase activity^16^,^17^. Emerging evidence has shown increased NLRP3 inflammasome activation and IL-1β responses in neutrophilic asthma and COPD^18^,^19^,^20^,^21^,^22^,^23^. Herein the anti-inflammatory efficacy of 17-oxo-DHA alone and in combination with the steroid fluticasone propionate (FP) was evaluated in LPS-stimulated peripheral blood mononuclear cells (PBMCs) from COPD patients and healthy individuals. Our goal was to assess (i) whether the PBMCs isolated from two study groups displayed different response to treatments; and (ii) whether externally administered 17-oxo-DHA was able to enhance the anti-inflammatory action of FP and, if so, to investigate the molecular mechanisms.

## Results

### 17-oxo-DHA displays anti-inflammatory actions and additive effects with FP in COPD patients and healthy individuals

**Figure 1:**
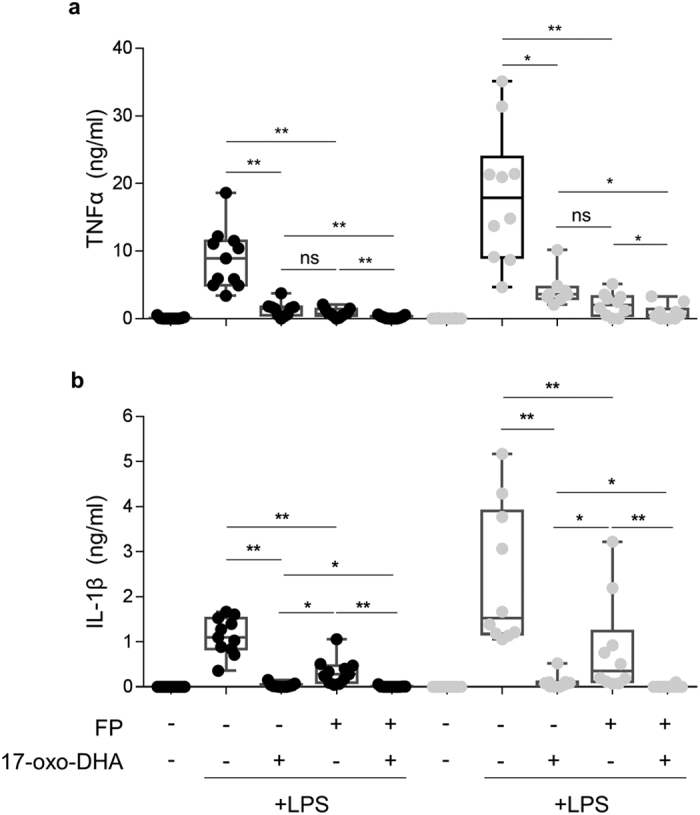
Modulation of LPS-induced TNFα and IL-1β release by 17-oxo-DHA alone or in combination with FP in PBMCs from COPD subjects and healthy individuals. Freshly isolated PBMCs from COPD patients (grey dots, N = 10) and healthy individuals (black dots, N = 11) were stimulated with 1 μg/ml LPS for 18 h. Where indicated, 17-oxo-DHA (5 μM) or FP (10 nM) were added, alone or in combination, 1 h before LPS. TNFα (**a**) and IL-1β (**b**) were measured in supernatants by ELISA. Data are presented in Box and whiskers plots with individual data points. The box extends from the 25th to 75th percentiles and the line in the middle of the box is the median. The whiskers represent the minimum and the maximum values. *p-value < 0.05; **p-value < 0.005; ns, p-value > 0.05.

**Figure 2:**
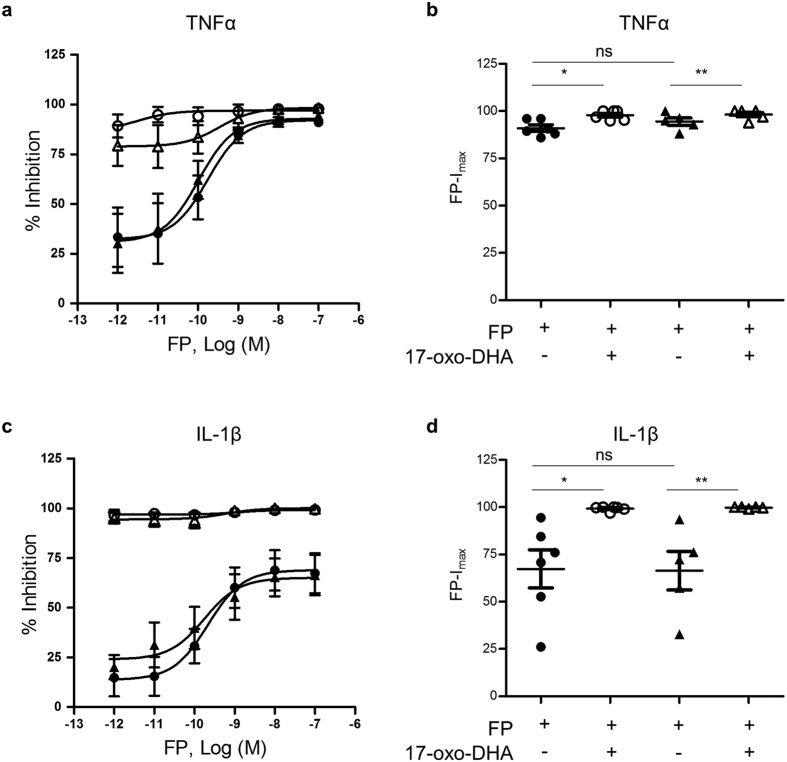
Effect of 17-oxo-DHA on FP-I_max_ of LPS-induced TNFα and IL-1β release in PBMCs from COPD patients and healthy individuals. (**a,c**) Freshly isolated PBMCs were treated with increasing concentrations of FP or a combination of FP and 5 μM 17-oxo-DHA for 1 h, then stimulated with LPS for 18 h. The levels of TNFα (**a**) and IL-1β (**c**) were measured in the supernatants by ELISA. Data are reported as % inhibition and are expressed as mean with SEM (N = 6 for the control group, N = 5 for the COPD group); (**b,d**) individual data reporting FP maximum inhibition (I_max_). ⚫, controls, FP; ○, controls, FP + 17-oxo-DHA; ▲, COPD, FP; △, COPD, FP + 17-oxo-DHA.

Freshly isolated PBMCs from moderate to severe COPD patients (N = 10) and healthy individuals (N = 11, control group) were treated for 1 h with 17-oxo-DHA, FP or 17-oxo-DHA + FP, then stimulated with LPS for 18 hours. Afterwards, TNFα and IL-1β concentrations were measured in the supernatants. The induction of TNFα and IL-1β release in response to LPS stimulation was significantly higher in PBMCs from COPD patients (median (range): 17.88 ng/ml (9.003; 23.91) for TNFα and 1.528 ng/ml (1.173; 3.901) for IL-1β) compared to PBMCs from healthy individuals (median (range): 8.92 ng/ml (4.96; 11.5) for TNFα and 1.096 ng/ml (0.8440; 1.528) for IL-1β). 17-oxo-DHA and FP, when administered alone, displayed a similar effect in suppressing TNFα release, while 17-oxo-DHA was more potent than FP in inhibiting IL-1β release (Fig. 1). The combination of 17-oxo-DHA and FP resulted in even greater suppression of TNFα and IL-1β release, showing additive effects in both study groups. To further investigate the impact of 17-oxo-DHA on FP actions, dose-response experiments were performed and the effect of 17-oxo-DHA on half maximal inhibitory concentration (IC_50_) and maximum inhibition (I_max_) of FP was evaluated (Fig. 2, Supplementary Figures S1, S2). No differences of IC_50_ and I_max_ were observed between the two study groups. When administered in combination with FP, 17-oxo-DHA significantly increased FP-I_max_ for both cytokines and in both study groups, without modifying FP-IC_50_.

### 17-oxo-DHA suppresses cytokine release by acting at transcriptional and post-transcriptional level

**Figure 3:**
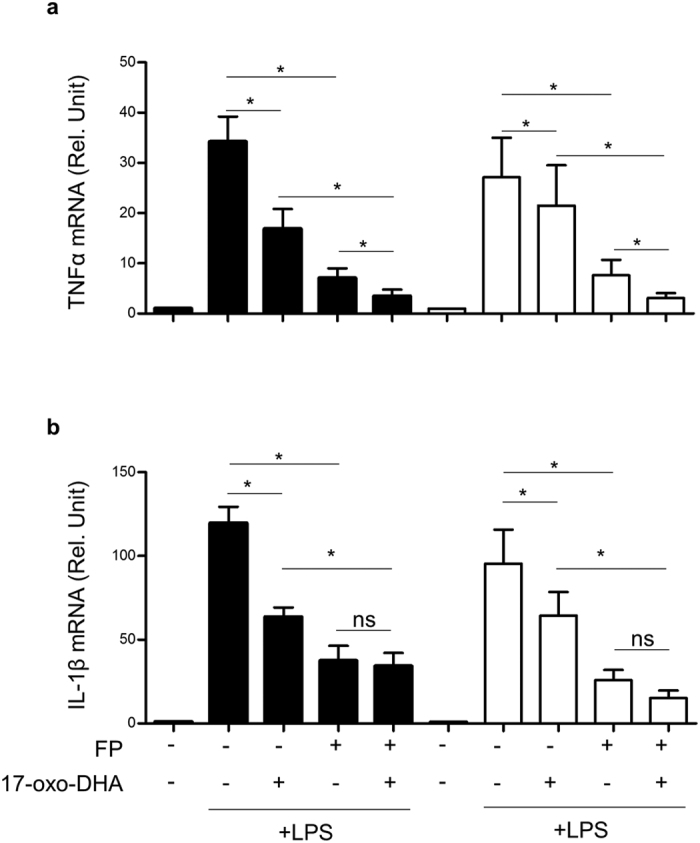
Modulation of LPS-induced TNFα and IL-1β mRNA expression by 17-oxo-DHA and FP alone or in combination in PBMCs from COPD patients and healthy individuals. PBMCs from COPD patients (empty bars, N = 6) and healthy individuals (filled bars, N = 6) were treated with 10 nM FP and 5 μM 17-oxo-DHA, alone or in combination, for 1 h then stimulated with 1 μg/ml LPS for 4 h. Total RNA was extracted and TNFα (**a**) and IL1-β (**b**) mRNA levels measured by RT-PCR. Mean with SEM are reported. *p-value < 0.05; ns, p-value > 0.05.

The effects of 17-oxo-DHA and FP, alone or in combination, on TNFα and IL-1β transcriptional modulation were evaluated (Fig. 3). Transcriptional inhibition of TNFα and IL-1β mRNA by 17-oxo-DHA and FP was similar in LPS-stimulated PBMCs from COPD patients and healthy individuals. 17-oxo-DHA and FP when given alone significantly suppressed the transcription of TNFα and, when given in combination, displayed additive effects, similarly to what observed when looking at the protein release profile. 17-oxo-DHA and FP inhibited LPS-induced IL-1β transcriptional activation. However, in contrast to the observed protein release profile, FP appeared to be stronger than 17-oxo-DHA in inhibiting IL-1β mRNA and no additive effects were observed between the two compounds. The release of mature IL-1β is regulated, among others, by the NLRP3 inflammasome. Since the expression of the NLRP3 protein itself is transcriptionally regulated, we evaluated the effect of 17-oxo-DHA and FP on the transcriptional modulation of NLRP3^24^. Although the differences were not statistically significant, both compounds reduced LPS-induced NLRP3 transcription to a similar extent (Supplementary Figure S3). This suggested that the strong inhibition of IL-1β protein release observed in response to 17-oxo-DHA was most likely due to post-transcriptional mechanisms of control. More specifically, we hypothesized that 17-oxo-DHA was able to block the release of mature IL-1β by inhibiting the NLRP3 inflammasome.

### 17-oxo-DHA, but not FP, suppresses IL-1β release by inhibiting the NLRP3 inflammasome

**Figure 4:**
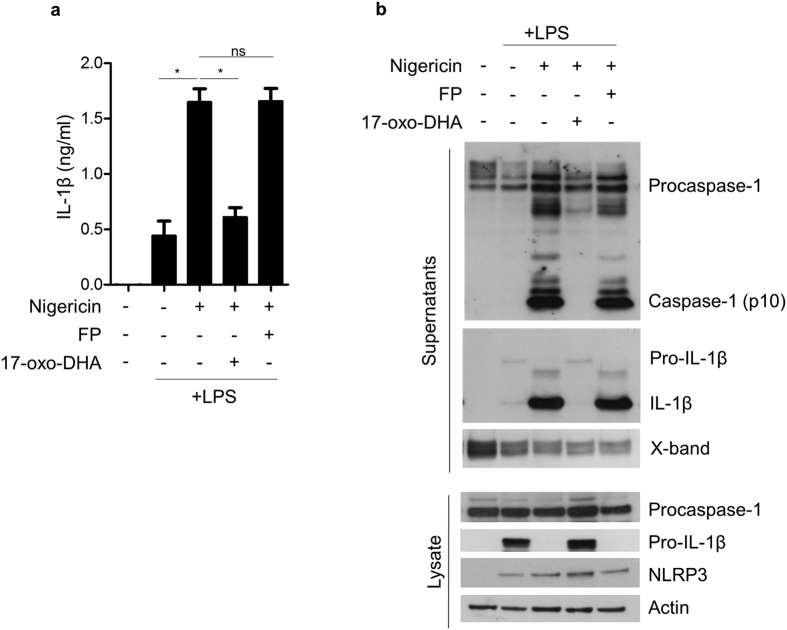
17-oxo-DHA, but not FP, suppresses NLRP3 inflammasome activation in PBMCs. PBMCs from healthy individuals (N = 6) were treated with 1 μg/ml LPS for 3.5 h, followed by FP (10 nM) or 17-oxo-DHA (5 μM) for 30 min, then by 10 μM nigericin for 30 min in serum-free medium. (**a**) IL-1β was measured in the supernatants by ELISA assay. Mean with SEM are reported. *p-value < 0.05; ns, p-value > 0.05. (**b**) Precursor and cleaved caspase-1 and IL-1β were measured by western blot in total cell extracts and supernatant precipitates, respectively. NLRP3 was measured by western blot in total cell extracts. A western blot representative of three independent experiments is shown.

**Figure 5:**
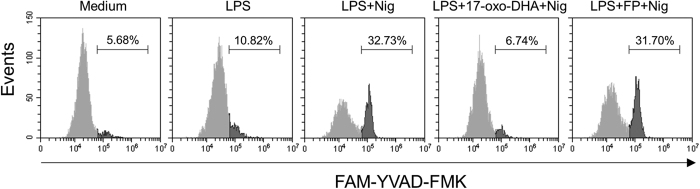
17-oxo-DHA inhibits caspase-1 activation in CD14^+^ cells. PBMCs from healthy individuals were treated with 1 μg/ml LPS for 3.5 h, followed by FP (10 nM) or 17-oxo-DHA (5 μM) for 30 min then by 10 μM nigericin for 30 min in serum-free medium. Cells were harvested, treated with a fluorescent probe recognizing active caspase-1 (FAM-YVAD-FMK), then stained for CD14. Analysis of caspase-1 activation was performed on CD14 positive cells. Representative histograms of three independent experiments are shown. Numbers indicated in the graphs represent the percentage of cells positive for active caspase-1.

To evaluate the effect of 17-oxo-DHA on NLRP3 inflammasome activation, LPS-primed PBMCs from healthy volunteers were stimulated with the NLRP3 inflammasome activator nigericin. The effect of 17-oxo-DHA on inflammasome activation was specifically evaluated by adding 17-oxo-DHA after LPS priming and before nigericin as previously reported^25^,^26^. The effect of FP on NLRP3 inflammasome activation was also evaluated. 17-oxo-DHA effectively inhibited nigericin-induced IL-1β release while FP had no effect (Fig. 4a). The actions of 17-oxo-DHA and FP on inflammasome activation were further assessed by evaluating the formation of cleaved caspase-1 and IL-1β as well as the expression of NLRP3, pro-IL-1β and procaspase-1. 17-oxo-DHA, but not FP, completely blocked the cleavage of pro-IL-1β and procaspase-1 (Fig. 4b). In the presence of nigericin, the strong effect on the extracellular release of IL-1β was accompanied with reduced intracellular levels of pro-IL-1β (Fig. 4b). The expression of the procaspase-1 did not show significant variations among different conditions while NLRP3 was induced by LPS, which is consistent with previous reports^27^. The inhibitory effect of 17-oxo-DHA on caspase-1 activity was further confirmed by flow cytometry using a fluorescent probe specific for active caspase-1. Double staining with fluorescent anti-human CD14 antibody was performed on LPS-primed and nigericin-activated PBMCs to evaluate the specific contribution of CD14^+^ monocytes to caspase-1 activation. Caspase-1 activation was inhibited by 17-oxo-DHA, but not by FP (Fig. 5).

### 17-oxo-DHA inhibits the activation of multiple inflammasomes as well as nigericin-induced pyroptosome formation and mitochondrial ROS generation

**Figure 6:**
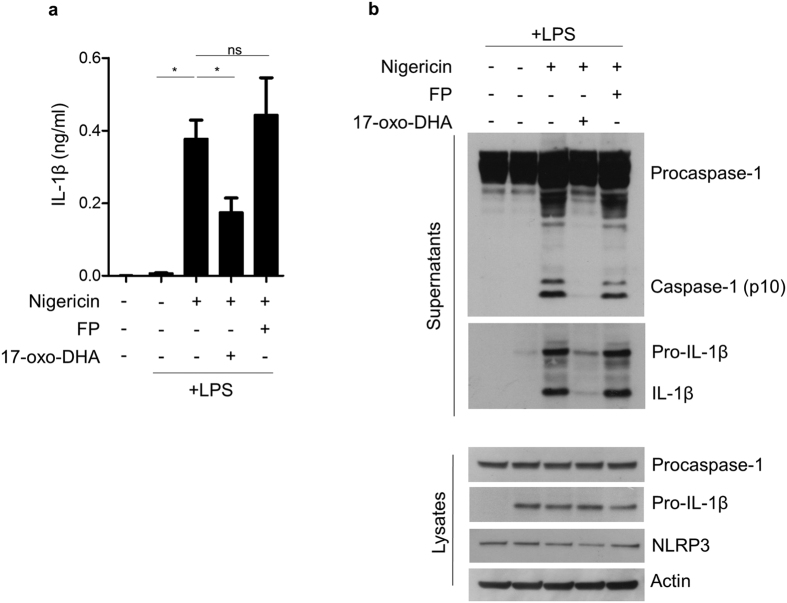
17-oxo-DHA, but not FP, suppresses inflammasome activation in THP-1 cells. THP-1 cells were treated with 1 μg/ml LPS for 3.5 h, followed by FP (10 nM) or 17-oxo-DHA (10 μM) for 30 min then by 10 μM nigericin for 30 min in serum-free medium. (**a**) IL-1β was measured in the supernatants by ELISA assay. Mean with SEM are reported. *p-value < 0.05; ns, p-value > 0.05. (**b**) Precursor and cleaved caspase-1 and IL-1β were measured by western blot in total cell lysates and supernatant precipitates, respectively. NLRP3 was measured by western blot in total cell lysates. A western blot representative of three independent experiments is shown.

**Figure 7:**
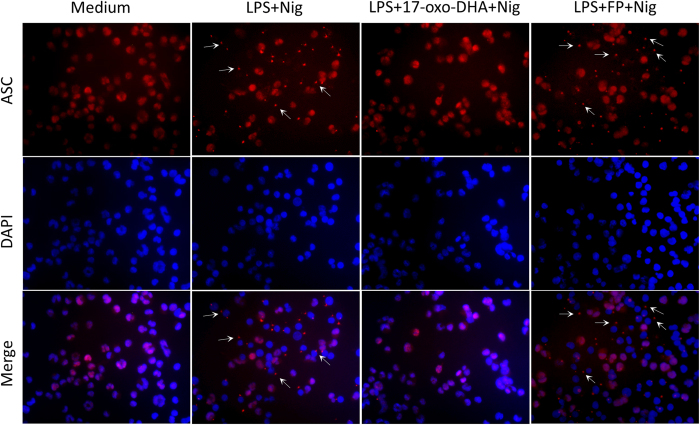
17-oxo-DHA inhibits NLRP3-mediated ASC pyroptosome formation. THP-1 cells seeded on glass coverslips were primed with PMA (0.5 μM) for 3 h. After 24 h, cells were stimulated with LPS for 3.5 h followed by nigericin in the absence or presence of 10 μM 17-oxo-DHA or 10 nM FP. ASC pyroptosome formation was assessed by immunofluorescence using anti-ASC antibody (red). Nuclei were stained with DAPI (blue). Arrowhead marks the ASC pyroptosome.

**Figure 8:**
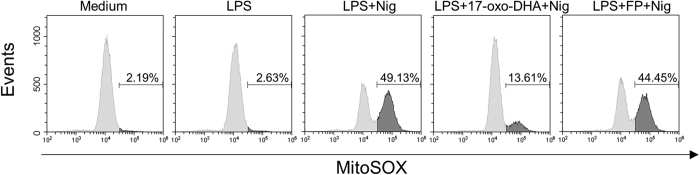
17-oxo-DHA, but not FP, reduces nigericin-induced mitochondrial ROS generation in THP-1 cells. THP-1 cells were treated with 1 μg/ml LPS for 3.5 h, followed by FP (10 nM) or 17-oxo-DHA (10 μM) for 30 min, then by 10 μM nigericin for 30 min in serum-free medium. Mitochondrial superoxide generation was measured by flow cytometric analysis after staining the cells with the fluorescent probe MitoSOX red. Representative histograms of three independent experiments are shown. Numbers indicated in the graphs represent the percentage of cells positive for MitoSox staining.

To further investigate the molecular mechanisms of inflammasome inhibition by 17-oxo-DHA and consistent with the activation of the inflammasome in peripheral blood monocytes, our analysis was expanded using the human monocyte-like cell line THP-1. The strong inhibitory effect of 17-oxo-DHA, but not FP, on nigericin-induced release of IL-1β, and cleavage of procaspase-1 and pro-IL-1β was confirmed (Fig. 6). Similar to PBMCs, the expression of procaspase-1 did not vary among conditions while pro-IL-1β was induced by the LPS treatment. The action of 17-oxo-DHA on NLRP3 inflammasome activation was confirmed also in response to ATP and MSU crystals (Supplementary Figure S4). Stimulation of LPS-primed THP-1 cells with nigericin induces the formation of the pyroptosome, a large supramolecular assembly of ASC that is believed to mediate caspase-1 activation^28^. Immunostaining for endogenous ASC in THP-1 cells revealed that 17-oxo-DHA, but not FP, strongly inhibited nigericin-triggered ASC pyroptosome formation (Fig. 7). Since the activation of the NLRP3 inflammasome is mediated by mitochondrial reactive oxygen species (ROS)^29^, we investigated the effect of 17-oxo-DHA on mitochondrial ROS generation. As reported in Fig. 8, 17-oxo-DHA, unlike FP, inhibited nigericin-induced mitochondrial ROS generation. We also tested the effects of 17-oxo-DHA on other inflammasome complexes. We found that 17-oxo-DHA significantly inhibited the activation of AIM2 and NAIP-NLRC4 inflammasomes induced by dA-dT and flagellin from S. typhimurium, respectively (Supplementary Figure S5). In addition, we performed a colorimetric assay using human recombinant caspase-1 to test whether the effects of 17-oxo-DHA were mediated by inhibition of caspase-1. As reported in Supplementary Figure S5, 17-oxo-DHA was able to inhibit caspase-1 activity.

### 17-oxo-DHA, but not FP, inhibits inflammasome-dependent GR degradation

**Figure 9:**
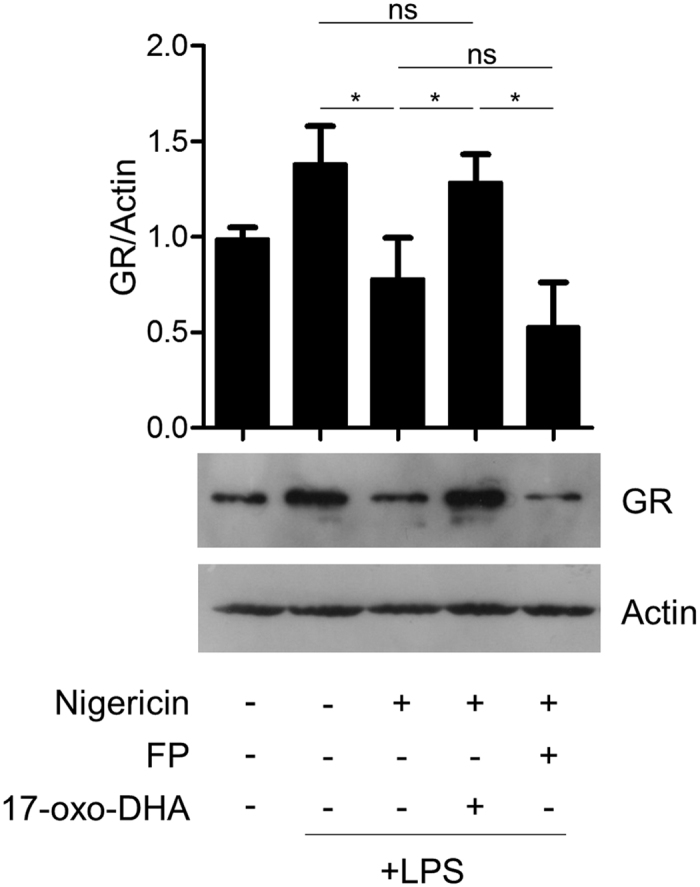
17-oxo-DHA, but not FP, inhibits inflammasome-dependent degradation of the glucocorticoid receptor. THP-1 cells were treated with PMA for 48 h then stimulated with 1 μg/ml LPS for 3.5 h, followed by FP (10 nM) or 17-oxo-DHA (10 μM) for 30 min, then by 10 μM nigericin for 30 min in serum-free medium. Expression of glucocorticoid receptor was measured in total cell lysates. Densitomeric analysis (N = 5) and a representative western blot image from five independent experiments are reported.

Inspired by a recent article showing that steroid-resistance in leukemia cells is caused by caspase-1-dependent cleavage of the GR^30^, we hypothesized that the activation of the NLRP3 inflammasome in THP-1 cells could lead to a reduction of GR levels. Data reported in Fig. 9 show that nigericin-dependent activation of the NLRP3 inflammasome significantly reduces GR levels. Interestingly, 17-oxoDHA, but not FP, inhibited nigericin-dependent degradation of GR and restored the receptor levels back to baseline.

## Discussion

We report that LPS-stimulated PBMCs from COPD patients released significantly higher levels of TNFα and IL-1β compared to PBMCs from healthy individuals, in agreement with previous findings^31^,^32^. Nonetheless, LPS-induced increase of mRNA levels of TNFα and IL-1β was comparable between PBMCs of both groups. This indicated the activation of post-transcriptional mechanisms of control that have been observed in COPD, such as increased mRNA stability and translation^32^,^33^,^34^,^35^,^36^. 17-oxo-DHA and FP significantly inhibited LPS-induced release of IL-1β and TNFα and, when administered together, displayed additive effects. However, since the steroid alone was very effective in inhibiting the release of TNFα, the additive action of 17-oxo-DHA was more relevant when looking at IL-1β release compared to TNFα. Of interest, the trend of TNFα transcriptional modulation by 17-oxo-DHA and FP paralleled the protein release profile. This suggested that control over TNFα release by FP and 17-oxo-DHA occurred mainly at transcriptional level. A different response was observed when looking at IL-1β. In particular, FP appeared to be stronger than 17-oxo-DHA in inhibiting IL-1β mRNA transcription, while 17-oxo-DHA was stronger than FP in suppressing IL-1β protein release. These data supported that 17-oxo-DHA acted through transcriptional as well as post-transcriptional mechanisms of control and led to the identification of the NLRP3 inflammasome as specific target of 17-oxo-DHA. We report that 17-oxo-DHA suppressed the activation of NLRP3, AIM2 and NAIP-NLRC4 inflammasomes, nigericin-induced formation of pyroptosome, cleavage of procaspase-1 and pro-IL-1β and release of mitochondrial ROS. We also show that 17-oxo-DHA inhibited the NLRP3 inflammasome-dependent degradation of the glucocorticoid receptor. Finally, we report that FP was unable to inhibit inflammasome activation.

The increased release of TNFα and IL-1β in PBMCs from COPD patients support that systemic inflammation is relevant in COPD, even more since all recruited COPD patients were under inhaled steroid treatment. Our results suggest that COPD patients may respond to bacterial infections with the release of higher levels of inflammatory cytokines compared to healthy individuals. Despite the enhanced cytokine release in response to LPS, PBMCs from COPD patients and controls displayed similar sensitivity to FP. Although initially surprising, this was consistent with previous reports showing that the efficacy of steroids in suppressing LPS-induced cytokine production from alveolar macrophages was not reduced in COPD patients compared to healthy individuals^37^. Interestingly, since the sensitivity to FP was unchanged when comparing the two study groups and LPS-stimulated PBMCs from COPD patients released higher levels of IL-1β and TNFα, the concentrations of these cytokines remained higher in the COPD group compared to healthy individuals even upon treatment with FP. Furthermore, the TNFα-related dose-response curve showed that the effects of 17-oxo-DHA became more significant at FP concentrations ≤1 nM. These low concentrations are close to the levels that are achieved within the lungs upon inhaled FP administration^37^,^38^ thus supporting the efficacy of 17-oxo-DHA in a more clinically relevant setting.

We show that the strong effect of 17-oxo-DHA in suppressing the release of IL-1β was mainly due to the inhibition of the NLRP3 and other inflammasomes. In human monocytes, LPS triggers alternative activation of the NLRP3 inflammasome via TLR4-dependent signaling cascade leading to the gradual release of IL-1β^39^. However, classical activation of NLRP3 inflammasome employs a two-step activation mechanism. Firstly, TLR4-dependent response leads to transcriptional upregulation of pro-IL-1β and NLRP3. Secondly, NLRP3-dependent response leads to inflammasome activation^26^. To elucidate the impact of 17-oxo-DHA and FP on inflammasome activation, independent on their transcriptional effects, FP and 17-oxo-DHA were added after TLR4 stimulation by LPS and before inflammasome activation by several triggers. We show that 17-oxo-DHA, unlike FP, inhibits the activation of NLRP3 and other inflammasomes by acting at multiple levels, including nigericin-induced mitochondrial ROS generation and pyroptosome formation as well as caspase-1 activity. The effect of 17-oxo-DHA on mitochondrial ROS generation may be linked to the previously reported induction of the Nrf2-dependent anti-oxidant response by 17-oxo-DHA^1^,^2^. Accordingly, the activation of Nrf2 negatively regulates NLRP3 inflammasome activity^40^. The inhibition of caspase-1 activity by 17-oxo-DHA measured in an enzymatic *in vitro* assay was not very strong, reaching about 30% inhibition even at the highest 17-oxo-DHA concentration (50 μM). This suggested that 17-oxo-DHA interfered with other mechanisms upstream of inflammasome activation regulatory cascades. Inhibition of nigericin-induced pyroptosome formation by 17-oxo-DHA represented one of such upstream mechanisms.

Whether the higher IL-1β levels that we observed in LPS-stimulated PBMC from COPD subjects were due to increased inflammasome activation is currently under investigation. The fact that FP was not able to inhibit inflammasome activation may provide an explanation for the poor suppression of IL-1β release observed when FP was added prior to LPS stimulation. Such inhibition most likely resulted from the transcriptional modulation of the pro-IL-1β gene by FP. Interestingly, this may explain why IL-1β levels remain high in the lung of COPD subjects, even after treatment with inhaled steroid, and most likely correlates with neutrophilic inflammation^20^. In fact, inflammasome activation leads to a rapid recruitment of neutrophils and monocytes to the inflammation site. Increased inflammasome activity has been observed in steroid-resistant COPD and neutrophilic asthma^20^,^21^,^23^,^41^,^42^ and it has been associated with infection-associated exacerbation episodes^41^. Furthermore, airway IL-1β and systemic inflammation in COPD patients are associated with exacerbation risk^5^. Overall, data available so far point at the NLRP3 inflammasome as a key target for the development of novel anti-inflammatory drugs for the treatment of COPD. This is further supported by previous data showing that the GR is a substrate for caspase-1^30^. Accordingly, we report that nigericin-dependent activation of the NLRP3 inflammasome caused a significant reduction of GR levels and that 17-oxo-DHA, unlike FP, inhibited this event. Since the expression of GR in target cells is a determinant of steroids pharmacological action, inflammasome-dependent GR degradation may contribute to reduced responsiveness to steroids^30^,^43^. Decreased GR expression has been reported in the lung and in circulating lymphocytes of COPD patients and in PBMCs from steroid-resistant asthmatics^44^. Interestingly, recent reports have shown that glucocorticoids appear to enhance the activity and expression of inflammasome components, which may result in enhanced inflammasome-dependent GR degradation^47^,^48^. Further investigations will be required to better assess whether inflammasome activation contributes to the reduced response to steroids that is observed in COPD and other chronic airways diseases.

17-oxo-DHA is endogenously generated during inflammatory reactions via a Cox-2-dependent enzymatic process^1^. We have previously shown that the levels of bioactive electrophilic derivatives of omega-3 PUFAs generated by immune cells can be modulated by increasing dietary uptake of fatty acid precursors^49^. Notably, omega-3 fatty acids have been correlated with protective effects in a number of chronic diseases^50^,^51^,^52^. We hypothesize that part of the beneficial effects of omega-3 PUFAs may be mediated by electrophilic bioactive keto-derivatives, such as 17-oxo-DHA. At present, the role of omega-3 PUFAs in the progression of COPD is still unclear and several clinical trials are currently investigating this^53^,^54^,^55^. Our data support that 17-oxo-DHA is a promising lead compound for the development of new treatments for COPD. We suggest that the combination of 17-oxo-DHA with steroids may achieve higher therapeutic efficacy compared to steroids alone especially in those conditions where increased inflammasome activation may lead to reduced GR expression and steroid-resistant chronic inflammation.

## Methods

### Subjects

Eleven healthy subjects and ten moderate to severe ex-smokers (>15 pack-year) COPD patients as defined by GOLD (the Global Initiative for Chronic Obstructive Lung Disease, http://www.goldcopd.com/) were included in the study. Characteristics of recruited patients are reported in Table 1. Ex-smokers were defined as subjects who had stopped smoking by more than one year. All the COPD patients included in the study were classified on the basis of lung function test: FEV1/FVC < 70%; 40% ≤ FEV1 < 80% predicted and broncho-dilatation reversibility less than 12%. Since all COPD subjects experienced more than two disease exacerbations during the previous year, they were treated with inhaled corticosteroids plus long acting beta 2 agonists and anti-cholinergic drugs. The following variables at admission were recorded: age, gender, smoking habits (Table 1). The study was approved by the Ethics Committee of Policlinico-Giaccone Hospital-Palermo Italy (authorisation reference number 7/2013) and was in accordance with the Helsinki Declaration. Informed written consents from the recruited patients were obtained.

### Reagents

17-oxo-4Z,7Z,10Z,13Z,15E,19Z-docosahexaenoic acid (17-oxo-DHA, ≥98%) was purchased from Cayman Chemical. The following chemicals were purchased from Sigma-Aldrich: phorbol 12-myristate 13-acetate (PMA), lipopolysaccharides from Escherichia coli 0111:B4 (LPS), nigericin sodium salt, fluticasone propionate (FP), adenosine 5′-triphosphate disodium salt hydrate (ATP) and poly(deoxyadenylic-thymidylic) acid sodium salt (dA-dT). Monosodium urate (MSU) crystals and Flagellin (Salmonella typhimurium) were from Adipogen. Lipofectamine^®^ 2000 Transfection Reagent was purchased from Thermo Fisher Scientific.

### THP-1 cell line, peripheral blood mononuclear cells (PBMCs) isolation and treatments

The human monocytic cell line THP-1 (ATCC TIB-202, kindly provided by Dott. Angelo Sala) was used in this study. THP-1 cells were grown in complete RPMI 1640 medium supplemented with 10% FBS and maintained in a humidified atmosphere of 5% CO_2_ in air at 37 °C. When THP-1 cells were treated with 17-oxo-DHA, a concentration of 10 μM was selected as the concentration giving maximal anti-inflammatory efficacy (measured as TNFα suppression) with no toxicity (data not shown). PBMCs were isolated from recruited patients or healthy volunteers using Ficoll-Paque density centrifugation, resuspended in complete RPMI 1640 medium supplemented with 1% FBS or no serum (as indicated) and stimulated for the indicated time. Dose-response experiments aimed at assessing the impact of 17-oxo-DHA on FP actions were performed on a subset of subjects (N = 6 for the control group, N = 5 for the COPD group). To define the optimal concentration of 17-oxo-DHA to be added in combination with increasing doses of FP, 17-oxo-DHA-IC_50_ and IC_25_ were calculated for TNFα suppression (Supplementary Figure S6). Then, the impact of 5 μM 17-oxo-DHA (between the 17-oxo-DHA-IC_50_ and IC_25_ value) and of the lower concentration of 2.5 μM was evaluated in combination with increasing doses of FP. Addition of 2.5 μM 17-oxo-DHA did not result in additive effects between the two compounds (Supplementary Figure S7). When 5 μM 17-oxo-DHA was added, additive effects were observed throughout the dose-response curve. Therefore, this concentration was selected for further experiments. All subjects had given their written informed consent.

### Inflammasome activation

Human PBMCs or THP-1 cells were treated with LPS (1 μg/ml) for 3.5 h in complete RPMI without serum, followed by 30 min treatment with FP or 17-oxo-DHA at the indicated concentration, alone or in combination, then treated with the following stimuli: nigericin (10 μM, 30 min), ATP (5 mM, 30 min), MSU (150 μg/ml, 1 h), dA-dT (1 μg/ml + lipofectamin 2000 2 μl/ml, 3 h), FLA-ST (10 μg/ml + lipofectamin 2000 2.5 μl/ml, 1 h). Where indicated, THP-1 cells were pretreated with 81 nM PMA for 48 h prior to LPS stimulation.

### Western blot

Supernatants were collected and cell pellets were resuspended in lysis buffer containing protease inhibitor cocktail (Roche, Basel, Switzerland). Cell lysates were stored at–20 °C o/n, then centrifuged at 13000 rpm for 10 min. Protein concentration was measured by Bradford assay. Supernatants were precipitated by adding an equal volume of methanol and 1:4 volume chloroform followed by 10 min centrifugation at 12000 rpm, RT. After removal of the upper layer, two-volumes methanol was added and samples were centrifuged again. The liquid phase was discarded, the pellet dried at 50 °C for 5 min then dissolved in reducing sample buffer, heated at 90 °C for 5 min then subjected to electrophoresis using 4–12% Criterion™ XT Bis-Tris Gels (Bio-Rad Laboratories) with MES buffer. For western blot analysis the following antibodies were used: antibody against IL-1β (AF-201-NA) was purchased from R&D Systems; antibody against caspase-1 (sc-515) was from Santa Cruz Biotechnology; antibody against NLRP3 (AG-20B-0014-C100) was from Adipogen. All secondary antibodies were purchased from Santa Cruz Biotechnology.

### Real-time PCR

Total RNA was extracted from PBMCs using TRIzol Reagent (Invitrogen) following the manufacturer’s instructions, and was reverse-transcribed into cDNA, using M-MLV-RT and oligo(dT)12–18 primer (Invitrogen). Quantitative real-time PCR of TNFα and IL-1β transcripts was carried out on Step One Plus Real-time PCR System (Applied Biosystems, Foster City, CA, USA) using specific FAM-labeled probe and primers (prevalidated TaqMan Gene expression assay for TNFα (Hs99999043m1), IL-1β (Hs01555410m1) and NLRP3 (Hs00918082m1), Assays on Demand, Applied Biosystems). Gene expression was normalized to glyceraldehyde-3-phosphate dehydrogenase (GAPDH) endogenous control gene. Relative quantitation of gene expression was carried out with the comparative CT method (2-ΔΔCt) [30] and was plotted as relative fold-change compared to untreated cells that were chosen as the reference sample.

### ELISA

The concentration of TNFα and IL-1β in cell supernatants was determined using human ELISA kit from R&D systems (DY210–05 and DY201–05) following manufacturer’s instructions.

### Flow cytometric analysis

Active caspase-1 was detected by flow cytometry using the fluorescent FLICA probe FAM-YVAD-FMK (ImmunoChemistry Technology) following manufacturer’s instructions. Briefly, freshly isolated PBMCs were treated as indicated then stained with 1X FLICA probe for 30 min at 37 °C. Cells were washed and stained with RPE-conjugated anti-human CD14 (DAKO, R0864) following manufacturer’s instructions. For the measurement of mitochondrial superoxide, THP-1 cells were stained with MitoSox Red (Thermo Fisher Scientific) at 1 μM for 15 min at 37 °C. The analysis was performed using Beckman Coulter Cytoflex flow cytometer equipped with CytExpert software (Beckman Coulter).

### Immunofluorescence

THP-1 cells were seeded on glass coverslips, primed with PMA (0.5 μM) for 3 h and allowed to attach for 24 h as previously reported^28^. For inflammasome activation by nigericin cells were stimulated as described above. After stimulation, cells were fixed with 4% paraformaldehyde for 15 min, permeabilized with 0.1% Triton x-100 in PBS for 5 min and blocked in PBST containing 2% BSA and 3% rabbit serum for 1 h. The cells were then incubated with anti-ASC antibody (sc-22514R - Santa Cruz Biotecnology; dilution 1 to 15, overnight) and TRITC-conjugated secondary antibody (711-026-152, Jackson ImmunoResearch Inc; dilution 1 to 50, 1 h). DAPI was used to stain nuclei. The cells were analyzed using Axioskop 2 Zeiss fluorescence microscope (Heidelberg, Germany).

### Caspase-1 assay

The effect of 17-oxo-DHA on caspase-1 activity was measured by performing a colorimetric assay as previously reported^56^. Briefly, 1 U of human recombinant active caspase-1 (Enzo Life Sciences) was incubated at 37 °C with 200 μM of the chromogenic substrate Ac-YVAD-pNA (Enzo Life Sciences) and the change in absorbance at 405 nm was recorded after 2 h. Where indicated, 17-oxo-DHA was incubated with caspase-1 30 min prior to substrate addition.

### Statistics

Repeated measures ANOVA was used, where applicable, to test for the presence of significant differences between conditions. If p-value was <0.05, then the Wilcoxon signed rank test was used for comparison of data within a single study group. The Mann-Whitney test was applied for comparison between study groups. For the analysis of data from THP-1 cells the paired *t*-test was used. The difference was considered significant at p-value < 0.05. The IC_50_ values (the concentration of FP that produced 50% of the maximal inhibitory effect) were calculated from the dose-response curves by non-linear regression analysis using GraphPad Prism version 6.00 for Windows, GraphPad Software (San Diego California USA). The data were presented as mean ± SEM, unless otherwise stated.

## Additional Information

**How to cite this article**: Cipollina, C. *et al*. 17-oxo-DHA displays additive anti-inflammatory effects with fluticasone propionate and inhibits the NLRP3 inflammasome. *Sci. Rep*. **6**, 37625; doi: 10.1038/srep37625 (2016).

**Publisher’s note:** Springer Nature remains neutral with regard to jurisdictional claims in published maps and institutional affiliations.

## Supplementary Material

Supplementary Information

## Figures and Tables

**Table 1:** Patient demographics.

	Controls	COPD
n	11	10
Age	65.5 ± 7.8	74.2 ± 5.0
Gender (M/F)	4/7	5/5
Packs/year	—	47.3 ± 18.9
FEV1 (% predicted)	113.5 ± 13.5	56.8 ± 14.7
FEV1/FVC (% predicted)	107.8 ± 6.2	62.9 ± 6.3

FEV1, forced expiratory volume in one second; FVC, forced volume vital capacity.
