# Supplementary material for: 17-oxo-DHA displays additive anti-inflammatory effects with fluticasone propionate and inhibits the NLRP3 inflammasome
Source: Sci Rep. 2016 Nov 24;6:37625. doi: 10.1038/srep37625 (PMC5121625; doi:10.1038/srep37625)
Supplement: Supplementary Information [file srep37625-s1.pdf]

17-oxo-DHA displays additive anti-inflammatory effects with fluticasone propionate and inhibits the NLRP3 inflammasome.

Chiara Cipollina, Serena Di Vincenzo, Liboria Siena, Caterina Di Sano, Mark Gjomarkaj and Elisabetta Pace.

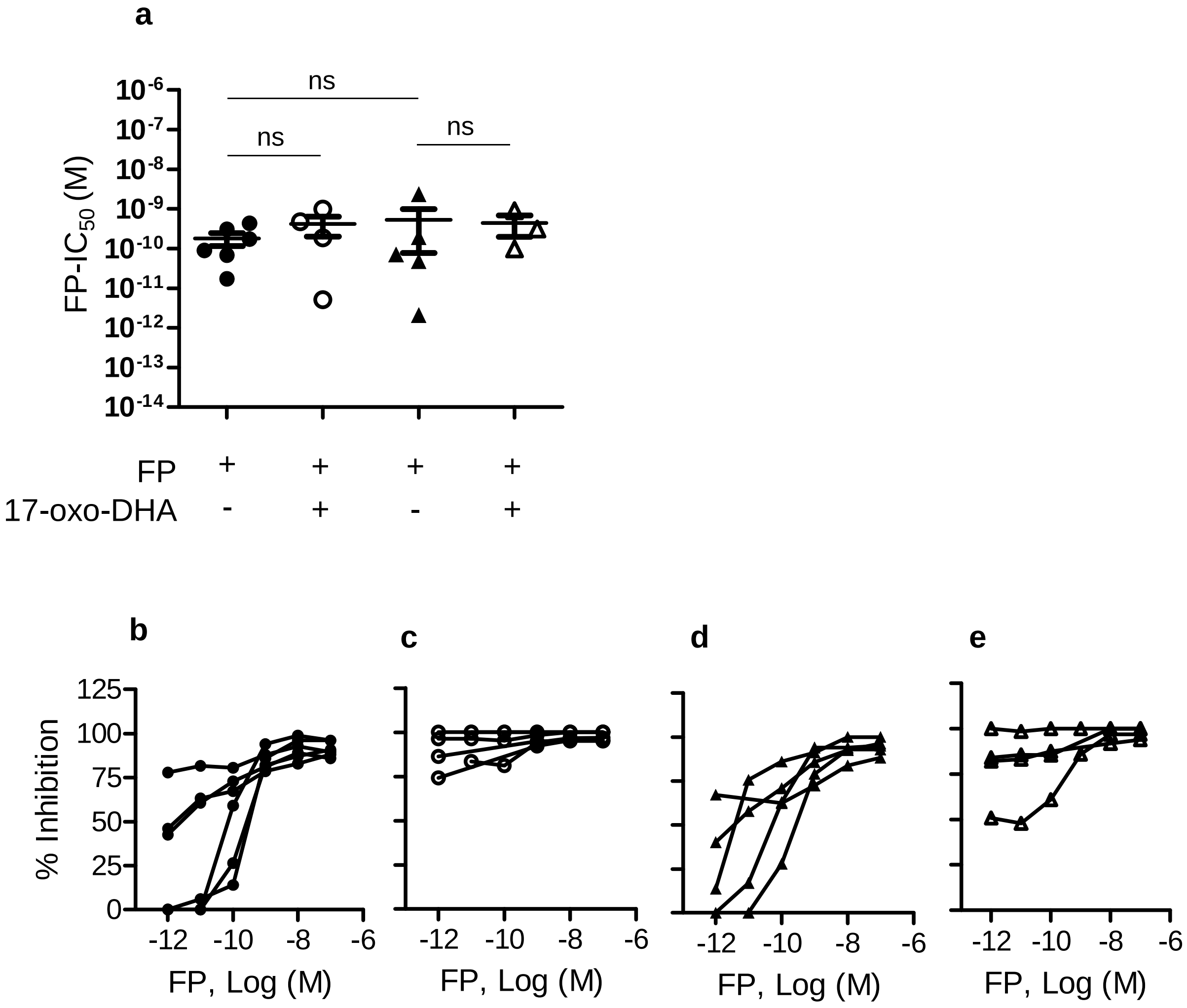

Figure S1. Effect of 17-oxo-DHA on FP-IC$_{50}$ of LPS-induced TNFα release in PBMCs from COPD patients and healthy controls and individual dose-response curves. PBMCs were treated with FP (10^{-12} to 10^{-7} M) or a combination of FP and 5 µM 17-oxo-DHA for 1h, and then stimulated with LPS for 18h. The levels of TNFα were measured in the supernatants by ELISA assay. (**a**) Individual values of IC$_{50}$ were calculated using the GraphPad software as the concentration of FP that produced 50% of the maximal inhibitory effect. In the samples where 17-oxo-DHA was added, it was not always possible to calculate IC$_{50}$. In these specific cases, these values were not reported. (**b-e**) Individual dose-response curves that were used to calculate IC$_{50}$ values. ●, controls, FP; ○, controls, FP+17-oxo-DHA; ▲, COPD patients, FP; △, COPD patients, FP+17-oxo-DHA.

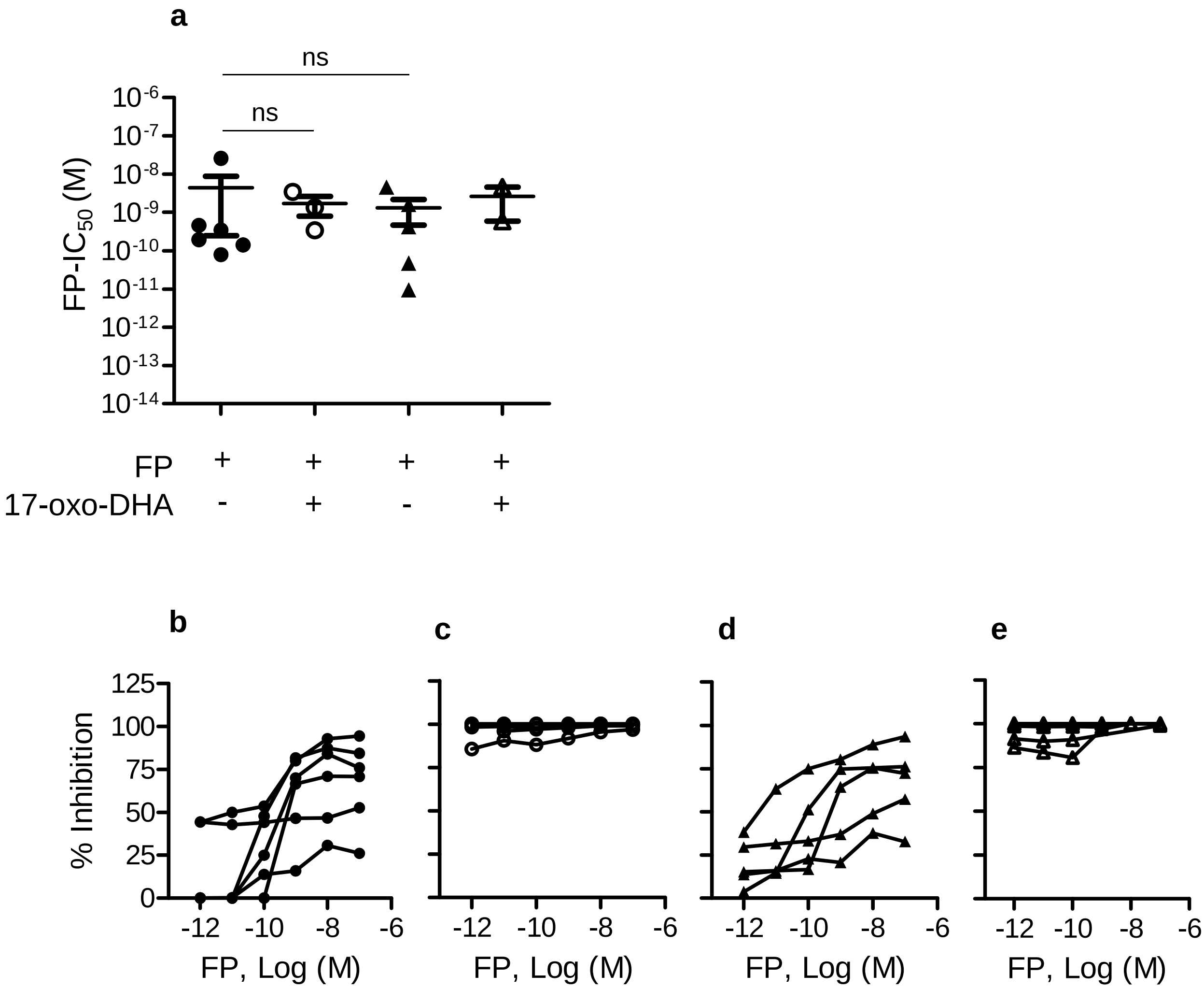

Figure S2. **Effect of 17-oxo-DHA on FP-IC$_{50}$ of LPS-induced IL-1β release in PBMCs from COPD patients and healthy controls and individual dose-response curves**. PBMCs were treated with FP (10^{-12} to 10^{-7} M) or a combination of FP and 5 µM 17-oxo-DHA for 1h, and then stimulated with LPS for 18h. The levels of IL-1β were measured in the supernatants by ELISA assay. (**a**) Individual values of IC$_{50}$ were calculated using the GraphPad software as the concentration of FP that produced 50% of the maximal inhibitory effect. In the samples where 17-oxo-DHA was added, it was not always possible to calculate IC$_{50}$. In these specific cases, these values were not reported. (**b-e**) Individual dose-response curves that were used to calculate IC$_{50}$ values. ●, controls, FP; ○, controls, FP+17-oxo-DHA; ▲, COPD patients, FP; △, COPD patients, FP+17-oxo-DHA.

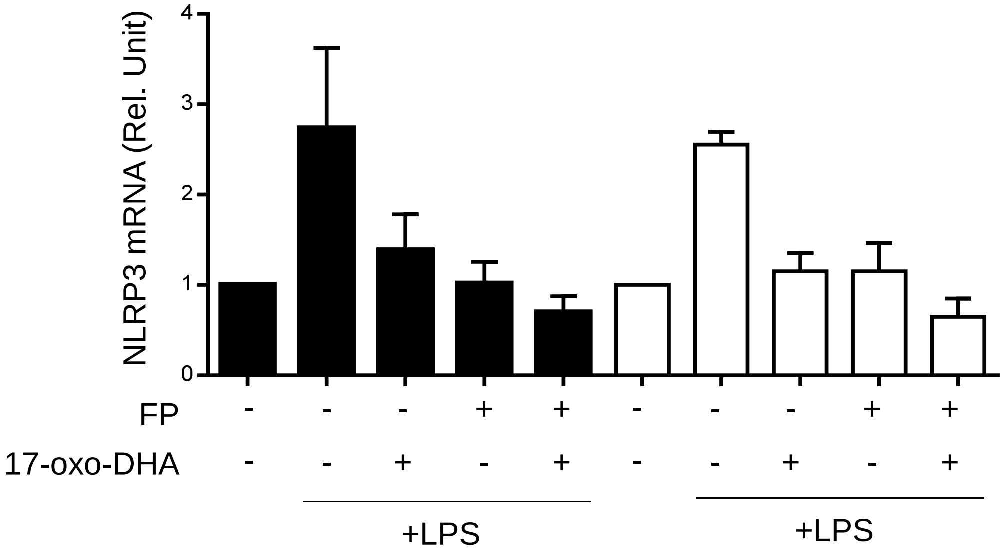

Figure S3. **Modulation of LPS-induced NLRP3 mRNA expression by 17-oxo-DHA and FP alone or in combination in PBMCs from COPD patients and healthy individuals.** PBMCs from COPD patients (empty bars, N=3) and healthy individuals (filled bars, N=3) were treated with 10 nM FP and 5 µM 17-oxo-DHA, alone or in combination, for 1h then stimulated with 1µg/ml LPS for 4 h. Total RNA was extracted and NLRP3 mRNA levels measured by RT-PCR. Mean with SEM are reported.

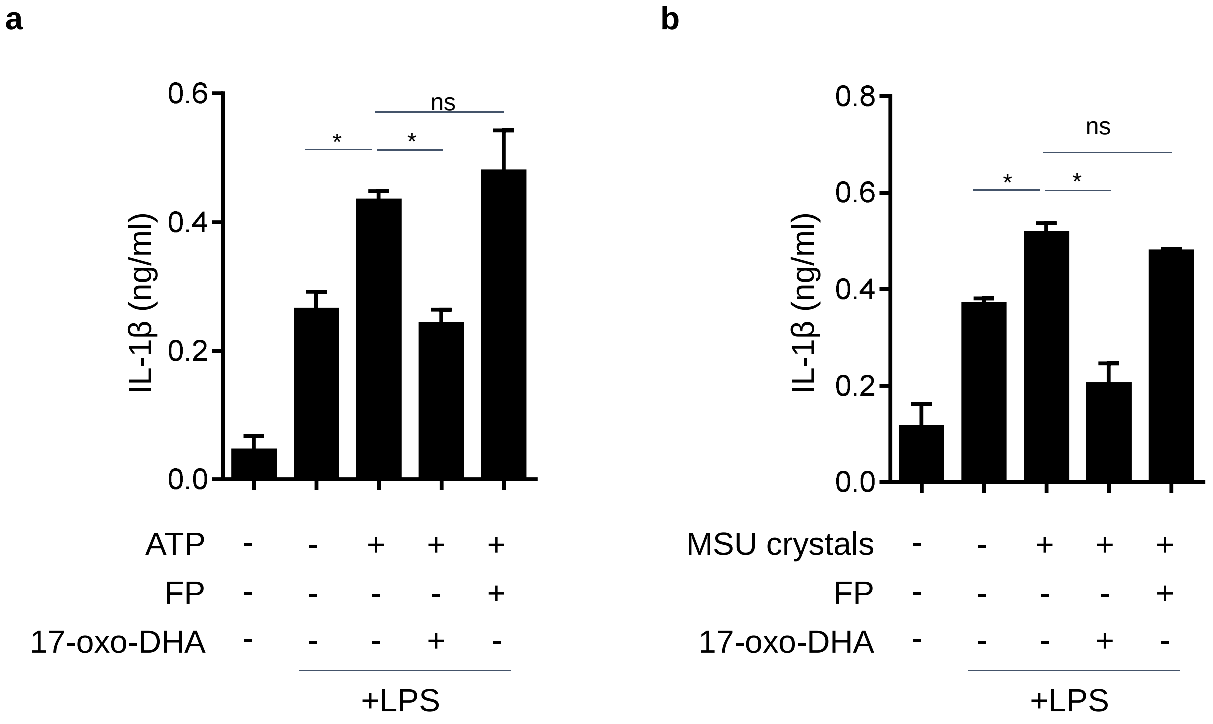

Figure S4. **Effect of 17-oxo-DHA on NLRP3 inflammasome activation by ATP and MSU crystals.** THP-1 cells were treated with 81 nM PMA for 48h then stimulated with 1 µg/ml LPS for 3.5 h, followed by FP (10 nM) or 17-oxo-DHA (10 µM) for 30 min then by 5 mM ATP for 30 min (a) or 150 µg/ml MSU crystals for 1h (b) in serum-free medium. IL-1β was measured in the supernatants by ELISA assay. Mean with SEM are reported. *, p-value <0.05; ns, p-value >0.05.

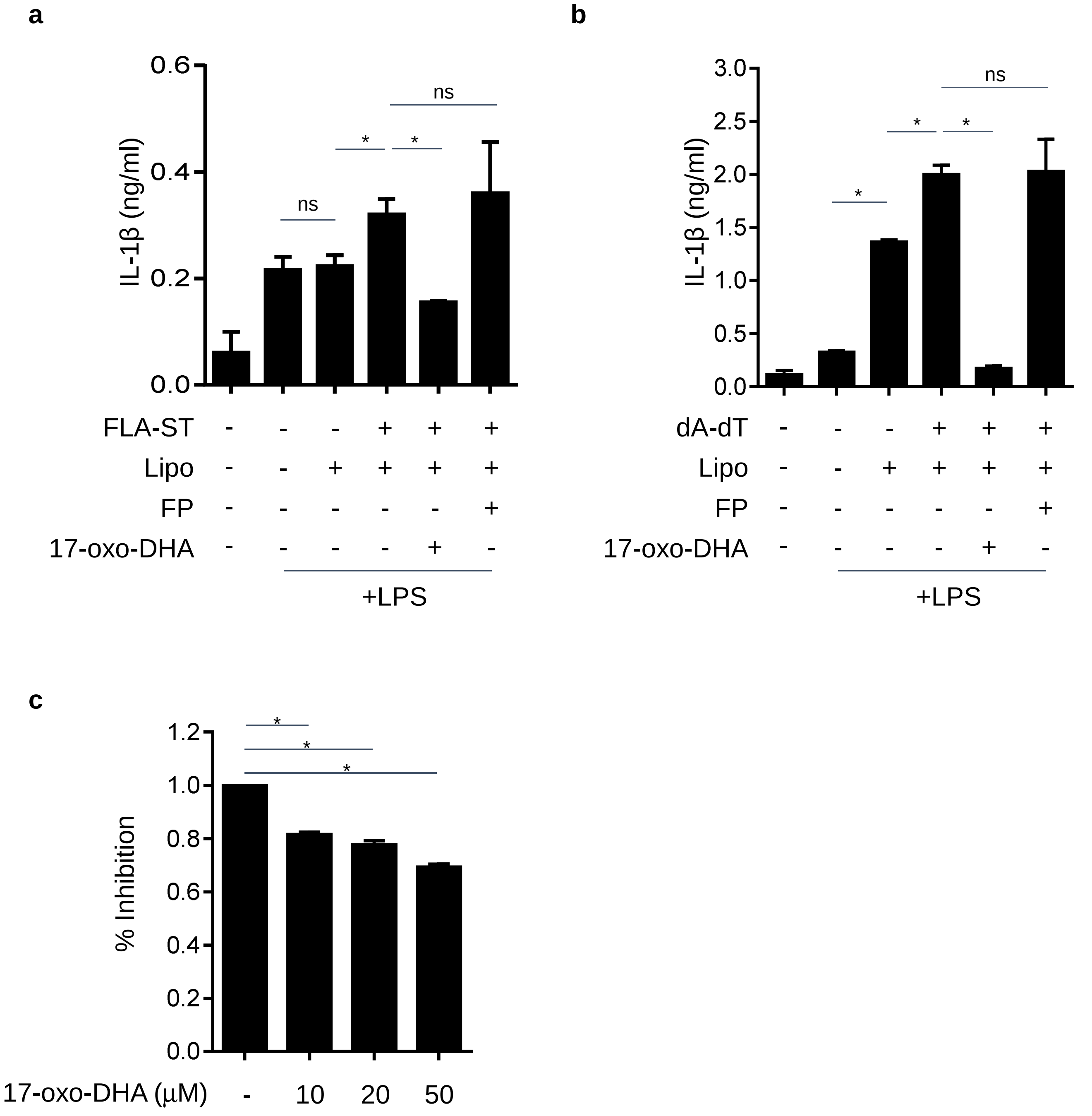

Figure S5. **Effect of 17-oxo-DHA on AIM2 and NLRC4 inflammasomes activation and on caspase-1 activity.** THP-1 cells were treated with 81 nM PMA for 48h then stimulated with 1 µg/ml LPS for 3.5 h, followed by FP (10 nM) or 17-oxo-DHA (10 µM) for 30 min then by 10 µg/ml flagellin from S. typhimurium (FLA-ST) for 1h (a) or 1 µg/ml dA-dT for 3h (b) in serum-free medium with lipofectamin 2000 (Lipo). IL-1β was measured in the supernatants by ELISA assay. (c) % Inhibition of caspase-1 activity by 17-oxo-DHA measured with an endpoint colorimetric enzymatic assay after 2 h. Mean with SEM are reported. *, p-value <0.05; ns, p-value >0.05.

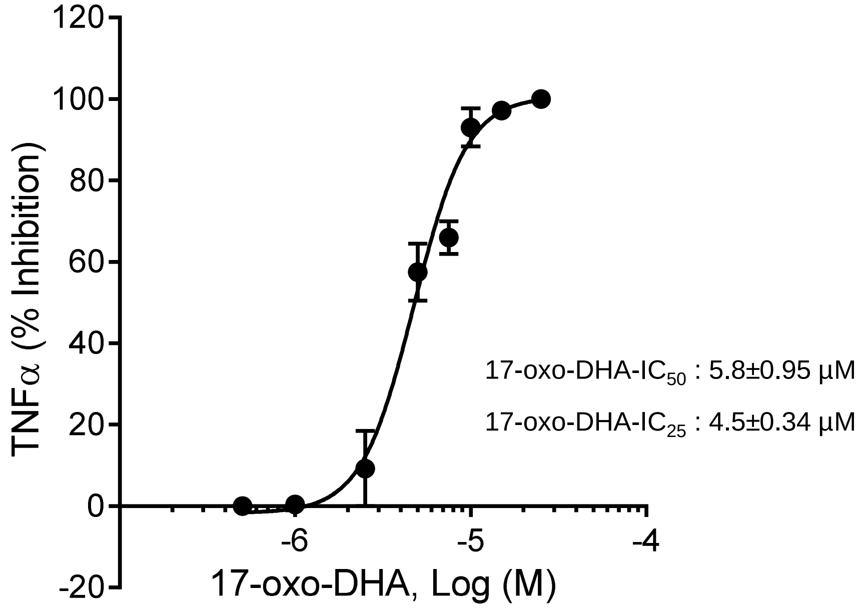

Figure S6. Effect of increasing doses of 17-oxo-DHA on TNFα inhibition. PBMCs isolated from healthy subjects were treated with increasing concentration of 17-oxo-DHA alone for 1h, then stimulated with 1 µg/ml LPS for 18h. The levels of TNFα were measured in the supernatants by ELISA assay. IC_{50} and IC_{25} values were calculated using the GraphPad software as the concentrations of 17-oxo-DHA that produced 50% and 25% of the maximal inhibitory effect, respectively. Data are reported as mean with SEM of 3 independent experiments.

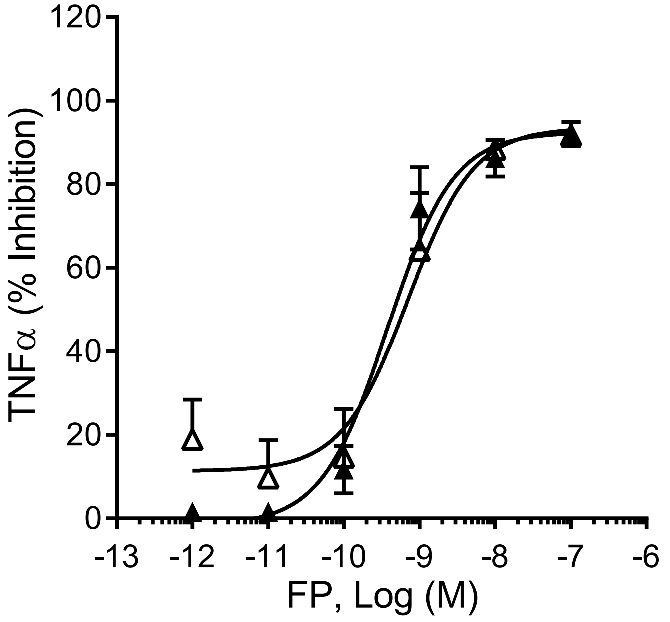

Figure S7. Effect of increasing doses of FP alone or in combination with 2.5 μM 17-oxo-DHA on TNFα inhibition. PBMCs isolated from healthy subjects were treated with increasing concentrations of FP alone (▲) or in the presence of 2.5 μM 17-oxo-DHA (△) for 1h, and then stimulated with LPS for 18h. The levels of TNFα were measured in the supernatants by ELISA assay. Data are reported as mean with SEM of 4 independent experiments (FP alone) or 3 independent experiments (FP+17-oxo-DHA).
